# Accreditation of private midwifery and nursing schools in Mali: a local sustainable solution to increasing the supply of qualified health workers

**DOI:** 10.1186/s12960-021-00654-4

**Published:** 2021-09-28

**Authors:** Cheick Oumar Touré, Sujata Bijou, Melanie Joiner, Andrew Brown, Jeanne Tessougué, Hamada Maiga, Fatoumata Dicko, Abdel Kader Keïta

**Affiliations:** 1 IntraHealth International, Bamako, Mali; 2 IntraHealth International, Chapel Hill, NC USA; 3 Gao Nursing School, Gao, Mali; 4 Mali Ministry of Health and Social Development, Bamako, Mali; 5 Ministry of Higher Education and Scientific Research, Malian Agency of Quality Assurance, Bamako, Mali

**Keywords:** Accreditation, WAHO, Quality, Private schools

## Abstract

**Background:**

The World Health Organization’s Global Strategy on Human Resources for Health (HRH) emphasizes the importance of dynamic and effective health worker regulation for achieving the health-related Sustainable Development Goals, with the establishment of education standards and quality assurance of education programs being critical. Governments in West Africa have struggled to address the problems within their higher education systems for health professionals, and it is now generally acknowledged that private institutions can play a crucial role in revitalizing the region’s outdated universities. However, the rapid expansion of private schools raises concerns about the quality of education and adequacy of regulatory mechanisms. The USAID-funded Mali HRH Strengthening Activity, led by IntraHealth International, assisted Mali’s Ministry of Health and Social Development to deliver targeted HRH interventions to improve the quality of education in private universities, better manage available health workers, and initiate a decentralized strategy for health worker recruitment and motivation.

**Case presentation:**

In 2018, the HRH activity leveraged the West African Health Organization (WAHO)’s accreditation system to support 10 private nursing schools to introduce WAHO’s regionally accepted, competency-based curriculum in reproductive, maternal, newborn, and child health. The project undertook a 10-step process to work alongside private nursing and midwifery schools to assess their current status against WAHO regional standards, implement action plans to address identified gaps, and support the institutions toward accreditation. As a result, eight schools in Mali are now accredited compared to only three at project inception.

**Conclusions:**

This case study underscores the importance of private school accreditation in Mali to improve the quality of health worker training through a standardized local curriculum. By supporting existing regulatory bodies that oversee accreditation, local capacity for initial accreditation of private nursing schools has been increased. Engaging universities in a partnership that shows the benefits of accreditation while maintaining a focus on the need to protect communities is critical to success. If the global community is to meet the WHO’s predicted health worker shortfall, then private education providers will need to be part of the solution. Robust and engaging health worker education accreditation systems are an essential part of that future.

## Background

### Understanding accreditation of health worker education

The World Health Organization (WHO)’s Global Strategy on Human Resources for Health (HRH): Workforce 2030 emphasizes the importance of dynamic and effective health worker regulation for achievement of the health-related Sustainable Development Goals (SDGs). The strategy points to the role of health worker regulation and accreditation of health professional education as necessary to ensure health workforce quality, manage numerical requirements, and better align health workforce investments with health system needs [1].

The WHO National Health Workforce Accounts (NHWA) describe health worker regulatory functions such as education standards, quality assurance, codes of conduct, scopes of practice, systems for licensure, professional development, and disciplinary measures as part of the “supply side” of health worker labor markets, which is balanced by the “demand side” (employment in the public and private sector), both of which need to be monitored and influenced by governments to ensure a strong health labor market [2]. WHO’s 2020 report on the state of the world’s nursing uses the NHWA approach to present global and regional data regarding the current workforce situation for nurses and midwives. The report highlights that “the world does not have a global nursing workforce commensurate with the universal health coverage and SDG targets” with Francophone West Africa having some of the lowest density of nurses globally (< 10 nurses/10,000 population). The report calls for strategic investment in nursing education, specifically noting an immediate need to “invest in the massive acceleration of nursing education—faculty, infrastructure and students—to address global needs, meet domestic demand, and respond to changing technologies and advancing models of integrated health and social care” [3].

Health outcomes for populations are linked to the supply of sufficient, competent, and appropriately skilled health workers. To address shortages in the supply of health workers, educational institutions need to increase their capacity to teach in terms of improved curricula and the numbers and quality of teachers and tutors, as well as physical infrastructure. Accreditation is an essential part of improving health training institutions—a key mechanism for assuring quality and protecting public health and safety. Additionally, having an accredited institution increases the number of student applications, thus expanding the pipeline of new health workers.

Accreditation is defined as the “action or process of officially recognizing someone as having a particular status of being qualified to perform a particular activity" with an example of “official certification that a school or course has met standards set by external regulators.” Accreditation of health worker education involves an external review of quality with four principal components: (1) it is based on written and published standards; (2) reviews are conducted by professional peers; (3) the process is administered by an independent body, and (4) the aim is to encourage organizational development [4]. Accreditation standards should be at an appropriate level to balance patient safety with the availability, accessibility, acceptability, and quality of health workers [5]. Ministries of health and education, or nominated professional councils, need to have both the legislative framework under which to establish health worker education standards and sufficient skills and resources to enforce accreditation in both private and public institutions.

Challenges in higher education in sub-Saharan Africa include declining quality standards, fiscal problems, poor faculty morale, outdated curricula, and rising unemployment among graduates. Dilapidated buildings, libraries, and laboratories exacerbate these challenges. Governments in West Africa have been unable to address the severe problems within their higher education systems, and it is now generally acknowledged that the privatization of higher education can play a crucial role in revitalizing the region’s outdated universities. Private universities offer a potentially viable alternative for expanding access to higher education in the region without incurring significant government costs [6]. However, the rapid expansion of private schools raises concerns about the quality of education, with regulatory mechanisms often viewed as inadequate and/or corrupt [5].

### Role of the West African Health Organization

The West African Health Organization (WAHO) encompasses 15 countries in the region (Benin, Burkina Faso, Cape Verde, Cote d’Ivoire, The Gambia, Ghana, Guinea, Guinea Bissau, Liberia, Mali, Niger, Nigeria, Senegal, Sierra Leone, and Togo); its vision is to promote better health through regional integration. WAHO has highlighted improved pre-service education (PSE) at the heart of its strategic plan, with a focus on harmonizing the curricula, accreditation criteria, standards of practice, and codes of ethics for nurses and midwives across the region. WAHO has established accreditation standards and a national health school in most Francophone West African countries, with these schools acting as accredited centers of excellence for public and private sector schools.

WAHO’s approach to standardization focuses on ensuring a minimum entry to practice standard for nurses and midwives as well as enabling the professional migration of these health workers in the region. For example, a midwife trained in Mali will have similar competencies as one trained in Niger.

### Malian context

Within the Malian context, private sector academic institutions play a significant role in supplying nurses and midwives for the national health system. In 2008, according to the Human Resources Department of the MOHSD, 53% of the candidates admitted for national certification as superior health technicians (nurses and midwives) and 95% of candidates admitted for national certification as technicians (public health nurses, obstetric nurses) were trained by private institutions [7]*.*

The MOHESR and MOHSD have not been able to invest in public health schools in the north of the country due to a lack of capacity and funding; however, the government of Mali supports the development of private schools in the northern regions and contributes to the training of health professionals to improve performance. This approach is supported by the national public–private partnership policy to leverage private sector educational institutions for the benefit of the public sector [8].

The USAID-funded Mali HRH Strengthening Activity (2017–2020; $5.5 M), led by IntraHealth International, assisted the MOHSD and MOHESR in six regions to deliver targeted, rapid interventions to train more health providers through improving the quality of education in private universities, better manage available health workers through the iHRIS health workforce information system, and initiate a decentralized strategy for health worker recruitment and motivation to compensate for the low rate of retention of health workers in rural and hard-to-reach areas.

## Case presentation

Mali is one of the 57 countries identified by the WHO as experiencing an HRH crisis. According to WHO estimates, 23 skilled health workers (doctors, midwives, nurses) per 10,000 people are needed to provide basic health services, but in Mali this ratio is only 6/10,000 [9]. Health personnel are unevenly distributed, the most qualified health workers being concentrated in Bamako and in the regional capitals. Northern regions of Timbuktu, Gao, and Kidal have ratios of 4, 5, and 13 skilled health workers per 10,000 people, respectively, while Bamako has a ratio of 23/10,000.

The deficit in health workers is exacerbated by a 3% annual rate of population growth, and amplified by Mali’s politico-military crisis, with armed attacks affecting the north and center of the country. Northern Mali presents a more challenging context because of its difficult geographical access, harsh climate, and low socio-economic status of the population. In addition to the shortage of health workers, the quality and availability of health services affects health outcomes. In Bamako, for example, almost all (98%) of births take place with the assistance of a trained provider; however, this percentage is much lower in the Kidal region (25%). The percentage of women who have made at least four or more recommended antenatal care visits is on average 25% in the three northern regions (28% in Timbuktu, 37% in Gao, and 9% in Kidal) compared to 72% in Bamako [10].

The Malian government has tried several strategies to improve the retention of health workers in the northern regions, such as financial incentives (zone bonuses), creating a local training institute for health workers, and the granting of conditional scholarships. In these regions many of the nursing and midwifery PSE institutions have been using out-of-date curricula based on incomplete or ill-defined competencies, leading to insufficient training of students by teachers, tutors, and clinical preceptors. Schools also struggle with basic management and the ability to find, hire, and retain sufficient qualified clinical faculty. All these factors contribute to students dropping out, low student achievement on national examinations, and inability to provide quality health care upon graduation. The selection of students can also have an impact on deployment and retention in underserved areas after graduation.

Health training institutions in Mali are overseen by the Ministry of Higher Education and Scientific Research (MOHESR; *Ministre de l'Enseignement Supérieur et de la Recherche Scientifique*), which ensures that the public has access to competent health care providers (nurses, midwives, physicians, and other allied health workers). It also ensures that providers receive quality education that prepares them to provide safe, competent, and ethical care; are certified or licensed upon entry to professional practice; and maintain competencies throughout their active clinical careers. They collaborate closely with the Ministry of Health and Social Development (MOHSD; *Ministre de la Santé et du Développement Social*) to review technical aspects of the accreditation process and provide them a written report on the accreditation results.

As Mali’s policymakers increasingly emphasized local training of health workers and use of private sector education institutions, the need arose to support private institutions to improve their curricula and its delivery to enable health workers to learn and work effectively in their specific local environments. In 2018, in collaboration with national and regional decision-makers, IntraHealth leveraged the WAHO accreditation system to support 10 private nursing schools to introduce WAHO’s regionally accepted, competency-based curriculum in reproductive, maternal, newborn, and child health (RMNCH), in line with the regional standard.

Table 1 outlines the 10-step process undertaken by the HRH Strengthening Activity to work alongside private sector nursing and midwifery schools to assess their current status against WAHO’s regional standards (presented in Table 2), create and implement corrective action plans to address identified gaps, and support the institutions through to accreditation. For Stages 1 to 8 of the process IntraHealth International provided direct support to each school to undertake preparatory and self-reflection stages, while Stages 9 and 10 of the process were carried out by MOHSD and MOHESR representatives, with logistical support provided by IntraHealth International.

**Table 1:** Key steps in the accreditation process for private nursing schools in Mali

Stages	Description
**Stage 1:** Engage key stakeholders	This stage consists of three key steps: Mapping and identifying relevant stakeholders critical to accreditation Engaging these stakeholders and working with them to raise their awareness Building support and involving communities and opinion leaders
**Stage 2:** Conduct desk reviews of relevant documents and materials to understand accreditation issues	There are two key steps to complete this stage comprehensively: Desk review of each regulatory body’s accreditation processes, procedures, and requirements Desk reviews of relevant documents from individual health training institutions
**Stage 3:** Support self-assessment of schools to identify accreditation gaps	This includes developing appropriate assessment tools and using them to identify gaps, barriers, and bottlenecks. Institutions must meet standards for accreditation in five areas (see Table 2): Curricula Faculty and staff Facilities, equipment, supplies, and other resources Governance, fiscal, and administrative capacity Environment and partnership
**Stage 4:** Validate and disseminate self-assessment findings	This stage involves analyzing and validating the data collected and then disseminating the findings to key stakeholders
**Stage 5:** Establish school and regional/district accreditation committees	To ensure ownership and sustainability of the accreditation process, this stage involves building the capacity of accreditation committees to evaluate, review, and update their own plans and functionality
**Stage 6**: Develop costed accreditation operational plans	This stage involves holding a planning meeting with each school to agree on and prioritize accreditation gaps and issues; draft accreditation operational plans and timelines; clarify roles, responsibilities, and expectations of all stakeholders; ensure accreditation operational plans are costed; and develop and sign a memorandum of understanding
**Stage 7:** Support region/district to implement costed accreditation operational plans	This stage consists of three key steps: Advocacy to the region and regulatory bodies for costed plans Supporting regions to develop and implement program-specific interventions for accreditation Implementing a plan to increase the number of and quality of course tutors
**Stage 8:** Conduct mock accreditation exercise	This stage ensures that a school is prepared for the accreditation process and consists of three key steps: Conducting a mock accreditation exercise Inviting the regulatory body for an advisory visit Implementing recommendations
**Stage 9:** Invite the regulatory body for accreditation and resource verification visit	This stage focuses on the accreditation visit itself and consists of two key steps: Submitting a progress report Paying and preparing for the regulatory bodies’ accreditation team
**Stage 10:** Continuous monitoring and evaluation	The last stage consists of the national or regional accreditation committees conducting quarterly supportive supervision and monitoring of the implementation of the costed accreditation operational plans. This monitoring by the accreditation committee should continue even after accreditation has been awarded to ensure any issues are promptly identified and solved so that accreditation status is maintained

**Table 2:** WAHO accreditation standards, common deficiencies, and key interventions

Accreditation standard	Common deficiencies	Key interventions
**Curricula** This standard refers to the existence of vision and mission statements; philosophy, goals and objectives; course content and detailed curriculum; class schedules; assessment methods and procedures; and operational committees	Lack of standardized materials available for existing RMNCH curricula and outdated curricula (some of which have not been revised in over 10 years) Lack of formalized hands-on learning at practicum sites	Introduce and implement WAHO harmonized RMNCH curriculum (facilitation guides and student modules) Develop internship agreements between health training schools and health facilities to enable hands-on learning
**Faculty and staff** This standard encompasses required teaching staff (number/composition and qualifications) as well as continuing professional training and development plans	Practicum site teachers and preceptors lack teaching and coaching competencies and skills Preceptors lack the motivation to supervise health students Non-existent or poorly functioning academic committees in most health training schools	Train teachers and preceptors on WAHO harmonized curriculum and competency-based approach Initiate coaching stipends for preceptor supervision Set up academic committees in the health training schools and ensure review of action items from meeting minutes
**Facilities, equipment, Supplies, and other resources** This criterion stipulates the minimum requirements in terms of equipment and supplies needed to effectively operate the school offices, hospitals, classrooms, and competency labs	Lack of sufficient classrooms with demonstration equipment as well as under-resourced laboratory facilities Lack of adequate teaching equipment and materials needed for quality instruction (e.g., computers, high-speed Internet connection, video projectors, anatomic models) Lack of health school improvement plans to address gaps in school infrastructure, including competency labs Lack of systematic monitoring of plans to measure progress to address gaps	Create or improve clinical skills building and simulation laboratory facilities. *These labs allow students and supervisors to practice different skills, including labor and childbirth, managing postpartum hemorrhage, provision of family planning (IUD insertion and removal and implant insertion), newborn resuscitation, and pelvic examination* Improve clinical sites, competency labs, facilities, equipment, and sufficient number of supplies to meet the needs of students in both rural and urban settings Conduct quarterly monitoring of health school improvement plans, including competency labs
**Governance, fiscal, and administrative capacity** The standard includes verification of legal documents creating the institution, approval of programs by a professional regulatory body, and the existence of a strategic plan, ethics code, organizational chart, staff/student records, class schedules, and budget, as well as policies and procedures	Absence of organizational structure and clearly defined roles and responsibilities (e.g., organizational chart, job description) Absence of or outdated strategic plan Absence of accounting manual and systematic implementation of fiscal processes and procedures Lack of public display of health school creation legal documents	Develop clear management structures (e.g., administrative council, pedagogical committee) in each school Develop a strategic plan focused on the WAHO accreditation process Provide consulting services to build health school capacity on accounting processes and procedures Display law/decree/act creating the institution
**Environment and partnership** This standard includes school welcome and direction signs, overall school layout, participation in associations and unions as planned for the well-being of students as well as recreation and sports facilities and environmental sanitation and safety	*Due to the high prevalence of poverty in this region, many eligible students, especially women, are unable to attend school because their families cannot afford the school fees and costs of living*	Complete data analysis tool to expose barriers to female participation in PSE and recruitment after graduation including: Initiation of scholarship program for selected students in need (majority went to women) Creation of nurseries which provided onsite caregiving for students who were mothers Conducted advocacy to address issues of gender discrimination in health workforce development

In terms of improving the quality of PSE though WAHO’s harmonized curricula, a baseline accreditation assessment showed that although the curricula was implemented in 9 of the 10 schools; only 1 of the 10 had an accreditation committee. The assessment showed a lack of a reproductive health curriculum in 3 schools; a deficiency of teaching materials needed for quality instruction; an absence of, or poorly functioning, academic committees in most schools; no standardized materials for family planning/reproductive health curricula; and a lack of laboratory skills in the training institutions. Moreover, traditional PSE did not include interactive, practical sessions in which students could practice and apply clinical skills to master new competencies. A more detailed explanation of WAHO Accreditation Standards, common deficiencies, and key interventions is outlined in Table 2.

*Accreditation steps adapted from WAHO’s accreditation guide and the Women for Health (WFH) program’s “Supporting Health Training Institutions to regain, maintain and upgrade their accreditation status.”* [11]

**Fig. 1:**
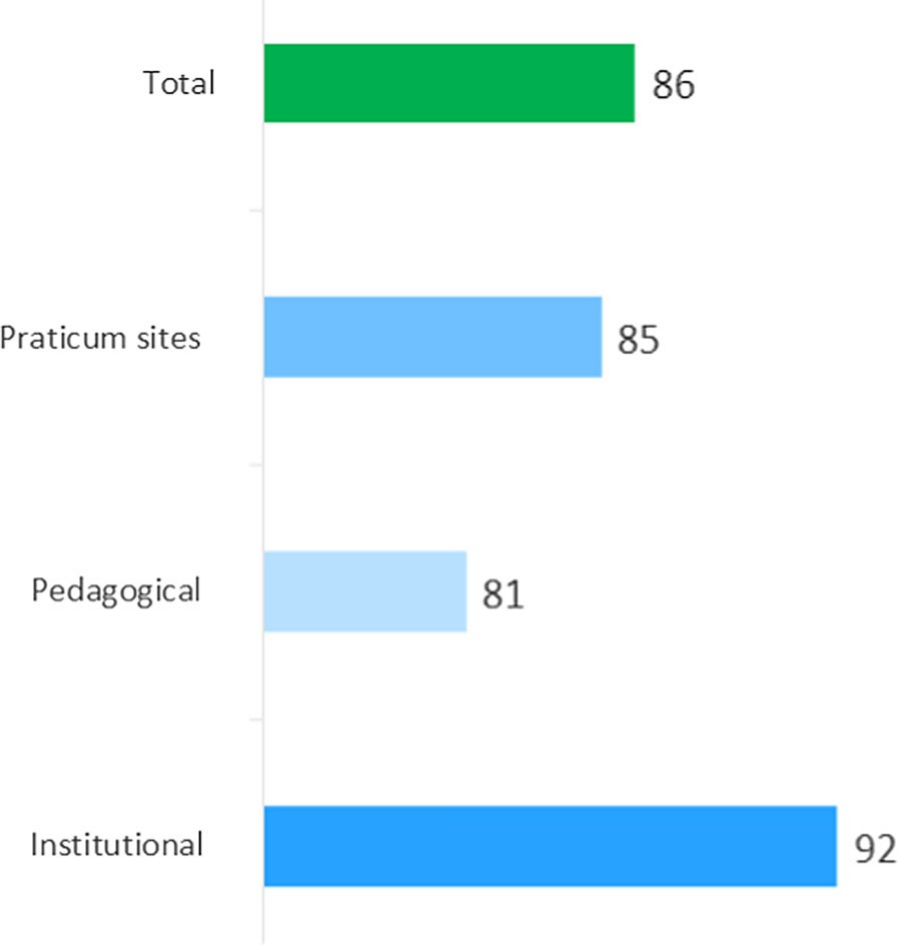
Final evaluation score (out of 100%) for one private nursing school

**Fig. 2:**
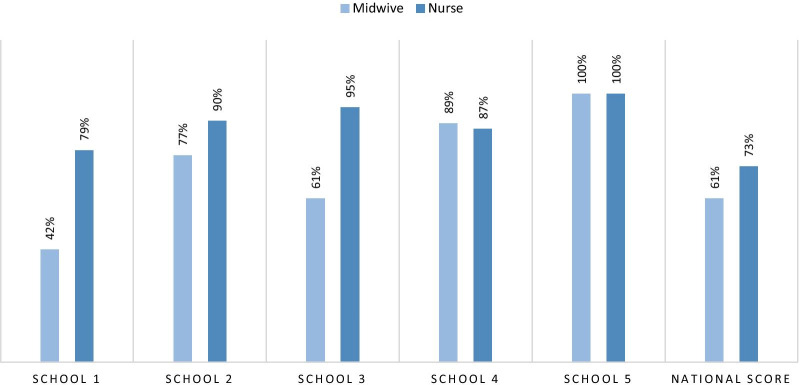
Graduation rates at schools applying the WAHO curriculum and the national average

As a result of these interventions, eight schools in Mali are now accredited and using the RMNCH competency-based approach (CBA) curriculum, compared to only three schools at project inception. Figure 1 provides the breakdown of the final accreditation score for one private institution with reference to the three accreditation modules: practicum sites, pedagogical, and institutional. A final score over 70% allows for 5 years of accreditation. Figure 2 highlights the graduation rates at six of the schools.

In 2019, a total of 2156 1st–3rd year students benefitted from CBA compared to only 1327 students at the beginning of the HRH activity in 2017, contributing to improved quality of the health workforce. In addition, 785/867 (90.5%) of students demonstrated the required competencies to progress to the next year level. A total of 203 students completed their clinical practice internships in rural and peri-urban areas within the northern regions.

At the Gao Nursing School, the pass rate on the national exam was 89% compared to a national rate of less than 50%. In addition, 93% of the graduates recruited by the MOHSD, communities, and nongovernmental organizations are still working in the North. As Dr. Mamadou Maiga, a teacher at Gao Nursing School, notes: “*Thanks to the competency-based approach implemented by the project to strengthen human resources for health, the student is now placed at the heart of his learning*.”

## Discussion and conclusions

This case study underscores the importance of private school accreditation in Mali in improving the quality of health worker training through the introduction of a standardized regional curriculum, leading to increased graduation rates and student enrollment. Private schools in particular are critical to filling the gaps that the public sector is unable to address in Mali. By supporting the existing regulatory bodies that oversee accreditation, local capacity for initial accreditation of private nursing schools has been increased. This will facilitate the initial accreditation of more schools and has established a sustainable process for ongoing quality reviews to maintain education standards.

However, we acknowledge that there are several administrative, financial, programmatic, and geographic limitations to implementing this approach. In order to participate, schools were required to have their legal documents in order and have access to a dedicated staff member (i.e., Dean and/or Director) to lead and oversee the accreditation process. In terms of financing, donor catalytic funding was needed to motivate schools to invest their own discretionary funding to implement the process effectively. From a program perspective, we noted that it was difficult for most schools to meet the ratio criteria of 10 students to 1 tutor. Lastly, there was difficulty identifying secure rural practicum sites within close proximity to schools. Insecurity also limits travel of accreditation teams to visit schools and can cause delays in the implementation process.

To institutionalize the process, Mali received substantial support from WAHO, USAID, and UNFPA to build the national accreditation system and to transfer skills to its national Center of Excellence (INFSS). Further, the establishment of an independent commission of national experts, responding to the MOHESR, guarantees the impartiality of recommendations. IntraHealth demonstrated success with the WAHO accreditation approach in Mali with catalytic funding from USAID, which led to increased government commitment and investment.

To sustain ownership of this process, accreditation committees in each school in Mali as well as at the regional level will be critical. Private schools have their own resources and will need to continue strengthening existing processes to improve quality. For example, continuing to build the capacity of accreditation committees to evaluate their own accreditation plans and functionality will help ensure that future operational plans are reviewed and updated on a regular basis. Ongoing self-assessment against WAHO standards (Table 2) is important because it can strengthen the cohesion and motivation of the teaching and administrative teams to stay focused on excellence and continuing improvement. The same criteria can be used for the opening of new training institutions.

It is important for Mali to continue to support and strengthen the accreditation mechanisms for better quality of health education. Accreditation covers both the curriculum and the institution that implements it and, in Mali, this also includes clinical internships. The existence of a well-defined legal status and the educational objectives for each institution standardized by WAHO are key achievements to sustain.

During the 75th Ordinary Session of the Economic Community of West African States (ECOWAS) Council of Ministers in December 2015 resolutions focused on a harmonized curricula for the training of health professionals and on the WAHO Strategic Plan 2016–2020, which dedicated an entire strategic objective to HRH focused on facilitating the training, use, and free movement of health professionals within the ECOWAS region. As a result, there is significant commitment and readiness among other countries to begin implementing this process in private health schools, especially given the significant contribution they provide to these countries’ overall workforce.

The WAHO accreditation process undertaken in Mali presents a proven method that could be replicated by other countries in the region that have a similar reliance on private sector education institutions for health workers. The establishment of WAHO nurse education accreditation standards and the strengthening of exemplar national health schools in many ECOWAS countries provides the basis for further sustainable development and harmonization of quality nurse education in the region. Such expansion could be a steppingstone toward greater regional cooperation across the nursing and midwifery workforce.

In recent years, several large-scale strategies are in the process of being implemented by WAHO to further support regional application. These include:

Development and implementation of harmonized curricula for initial health worker training and specialties.

Establishment of three Centers of Excellence for the issuance of master’s degrees at inFAS Abidjan, INSP in Niamey, and INFSS in Bamako.

Accreditation of training institutions.

Updating of the regional action plan for the motivation and retention of health workers.

Elaboration of the regional plan for HRH development and support to countries for its implementation.

Networking of health professional associations and bodies.

As we have seen with the COVID-19 pandemic, there is an increased necessity to shift technical leadership into the regions of countries to ensure strong anchoring and localization of the processes. In addition, there are opportunities to better leverage digital learning platforms and approaches within Mali and across the region, in order to offer greater flexibility for updated curriculum content (e.g., on COVID-19 and emerging diseases) and improve access for non-traditional students.

Supporting the accreditation of private universities delivering health worker education is an important element in strengthening the supply of sufficient health workers in countries that rely on the private sector for such education. Engaging universities in a partnership that shows the benefits of accreditation while maintaining a focus on the need to protect communities is critical to success. If the global community is to meet the 18 million health worker shortfall predicted by the WHO for 2030, then private sector education providers will need to be part of the solution. Robust and engaging health worker education accreditation systems are an essential part of that future.

## Data Availability

The datasets used and/or analyzed during the current study are available from the corresponding author on reasonable request.

## Abbreviations

CBA: Competency-based approach
HRH: Human Resources for Health
MOHESR: Ministry of Higher Education and Scientific Research
MOHSD: Ministry of Health and Social Development
NHWA: National Health Workforce Accounts
PSE: Pre-service education
RMNCH: Reproductive, maternal, newborn, and child health
SDGs: Sustainable Development Goals
USAID: United States Agency for International Development
WAHO: West African Health Organization
WHO: World Health Organization
